# Climate Change: Implications for the Yield of Edible Rice

**DOI:** 10.1371/journal.pone.0066218

**Published:** 2013-06-12

**Authors:** Xiangqian Zhao, Melissa Fitzgerald

**Affiliations:** Grain Quality and Nutrition Centre, International Rice Research Institute (IRRI), Metro Manila, Philippines; University of Florida, United States of America

## Abstract

Global warming affects not only rice yield but also grain quality. A better understanding of the effects of climate factors on rice quality provides information for new breeding strategies to develop varieties of rice adapted to a changing world. Chalkiness is a key trait of physical quality, and along with head rice yield, is used to determine the price of rice in all markets. In the present study, we show that for every ∼1% decrease in chalkiness, an increase of ∼1% in head rice yield follows, illustrating the dual impact of chalk on amount of marketable rice and its value. Previous studies in controlled growing conditions report that chalkiness is associated with high temperature. From 1980–2009 at IRRI, Los Baños, the Philippines, annual minimum and mean temperatures, and diurnal variation changed significantly. The objective of this study was to determine how climate impacts chalkiness in field conditions over four wet and dry seasons. We show that low relative humidity and a high vapour pressure deficit in the dry season associate with low chalk and high head rice yield in spite of higher maximum temperature, but in the opposite conditions of the wet season, chalk is high and head rice yield is low. The data therefore suggest that transpirational cooling is a key factor affecting chalkiness and head rice yield, and global warming *per se* might not be the major factor that decreases the amount and quality of rice, but other climate factors in combination, that enable the crop to maintain a cool canopy.

## Introduction

Rice is the most important source of calories for 50% of the world’s population. In the face of population growth and urbanisation, rice production must increase at rates unseen this century to feed the growing global population [1]. For the past three decades, the annual growth rate for rice production has decreased from 2.73%, in 1980s to 0.88% since 2000 [2]. The message is that overall yields must increase, or significant improvements made to minimize yield-reducing factors such as postharvest loss of grain. An additional challenge to increasing rice production is the effect of climate change, especially global warming, which decreases the yield of rice and increases factors that affect recovery of intact grains after polishing [3], [4].

The mean global air temperature has increased by 0.3 to 0.6°C over the past century [5]. Further, the average temperature for July 2010 was 0.55°C warmer than that from 1951–1980 [6]. Higher minimum temperature reduces the yield of rice and the mechanism is thought to be due to higher loss of carbon through increased respiration [7]. By contrast, higher maximum temperature has been shown to raise yield [4], presumably through improving accumulation of assimilates, lower respiration, higher tillering, leaf-area expansion, stem elongation, faster grain filling, and crop phonological development [3].The impact of high temperature on rice production is not only seen on yield but also on grain quality. High temperature during both the day and the night has been shown to increase chalkiness, which causes grains to break during dehulling and polishing, leading to a decrease in the amount of acceptable or marketable polished rice [8], negating any yield gain in the field. Coupled with urbanisation and shrinking farm size, new varieties will have to have both higher field yield of paddy rice and of the yield of whole grains of rice that remain after polishing (head rice yield, HRY), and a path to the latter is through understanding the factors leading to chalk, to enable breeding programs to target those factors in new climate-ready germplasm.

Many studies of the genetic and environmental effects on chalk indicate that heredity and climate conditions, especially high air temperature during the grain-filling period, are the most important factors affecting chalkiness [9], [10], [11], [12], [13]. However those conclusions were based on just a few varieties grown in controlled temperature conditions [12]. Such conditions often do not take account of other climate variables such as solar radiation, humidity and rainfall. In addition, it has been shown that a discrepancy exists between estimates of the effects of warmer temperatures on crop yields based on field conditions, versus those derived from modeling and experiments conducted under controlled conditions [14]. The objective of the present paper is to progress our understanding of the effect of climatic conditions on rice yields, by stepping beyond paddy yield and out of the glasshouse, to understand how different climate factors affect the quality, and therefore acceptability of rice, using a panel of 39 varieties grown in the field over eight seasons in tropical conditions.

## Materials and Methods

### Field Experiments

A set of 39 varieties of rice (*Oryza sativa* L.) developed by the International Rice Research Institute (IRRI), with wide variation in chalkiness and head rice yield (HRY), and small variation in grain length and shape, was used in this study (Table S1). Experiments were conducted at IRRI, Los Baños, Laguna, Philippines across 4 dry seasons (DS) (2005, 2006, 2007 and 2009) and 4 wet seasons (WS) (2005, 2006, 2007 and 2008). The same 39 varieties were grown in every season. Seeding date in the DS was mid-December and in the WS was the second or third week of June. In each growing season, seeds were sown in the seedling nursery and 21-day-old seedlings were transplanted with 2–3 seedlings per hill. Plots were 8 m^2^ (1.6 m×5 m) and each consisted of 200 plants with a spacing of 20 cm between plants and 20 cm between rows. Fertilizer of 40 kg N ha^−1^, 40 kg P ha^−1^, and 40 kg K ha^−1^ were applied basally. Forty kg N ha^−1^ was top-dressed at 2 weeks after transplanting. Fields were flooded 3 days after transplanting, and a water depth of 3–5 cm was maintained until 7–10 days before harvesting. Insects, diseases, and weeds were controlled by using approved pesticides to avoid potential effects on grain quality. Flowering dates for each season were kindly provided by the Plant Breeding, Genetics and Biotechnology (PBGB) division; flowering date was the date that 80% of plants were heading in each plot. All varieties were harvested at 30 days after flowering (DAF).

### Quality Evaluation

Grains from the middle rows in each plot were harvested for laboratory analysis. Freshly harvested paddy was dried to between 12 and 14% moisture content, and equilibrated in paper bags at 25°C for 3 months before further analysis. For determining HRY, 125-g rough rice from each plot was dehulled (Satake Engineering Co., Japan), and the resulting brown rice polished (Grainman Machinery MFG. Corp., Miami FL, USA). Head rice was separated from broken grains using a shaking sieve. HRY was determined as the percentage of intact, unbroken grains of rice relative to the original 125 g of paddy. Physical traits such as chalkiness, grain length (GL) and grain width (GW), of the polished grains were measured using a Cervitec Grain Inspector 1625 (Foss, Denmark). The percentage of grains with chalkiness (PGWC) was determined manually using the images from the Grain Inspector.

### Weather Data

The weather station and methods of data collection have been fully described previously [3]. Solar radiation (MJ/m^2^), sunshine (hrs), rainfall (mm), evaporation (mm), air temperature (maximum and minimum temperature, °C) and RH (%) were measured using a Sunshine Recorder (Gunn Bellani Radiation Integrator, Campbell Stokes), Class A evaporation pan, Rainguage, Alcohol-in-Glass Thermometer and Hygrothermograph respectively. Vapor pressure deficit (VPD, kPa) was calculated from temperature and RH. Diurnal variation was defined as the difference between maximum and minimum temperature. Weather data for each day during every growing season were kindly provided by the Climate Unit of the IRRI, which conforms to the World Meteorological Organization standard specifications.

### Data Analysis

In order to determine associations between climates parameters from 1980–2009, average annual data was analysed using Pearson’s correlation coefficients. The 5-days average value is a useful unit in dealing with meteorological phenomena, especially if the data have to be relevant to agriculture. In addition, grain filling was divided into stages of initial filling, milky, waxy, yellow and mature corresponding to 0–5, 6–15, 16–20, 21–25 and 26–30 DAF respectively. To minimize fluctuations in weather data during grain-filling across the 8 seasons, 5-day averages values were calculated (0–5, 6–10, 11–15, 16–20, 21–25 and 26–30 DAF) and then the average over the 39 varieties was used for comparison and analysis. Differences between all climate parameters, grain quality traits in the DS and WS were examined using the Student’s t-test. The relationships between grain quality traits and climate parameters of each variety and the average of the 39 varieties were evaluated using Pearson’s correlation analysis by SPSS 16.0 (SPSS Inc.).

## Results

### Weather Patterns at IRRI from1980–2009

Over the years from 1980–2009, the annual minimum and mean temperatures increased significantly, by 1.21°C and 0.80°C respectively, whereas the maximum temperature increased by 0.40°C but this was not significant (Table 1). The diurnal variation (max.-min.) showed a significant decrease −0.82°C (Table 1). VPD and RH changed significantly. No significant changes were observed in the annual average for other parameters.

**Table 1:** Trends in climatic parameters from 1980 to 2009 and observed average data of 8 seasons.

Parameters	30 years			8 seasons		
	Equation	R-squaredvalue	Expected variation	4 DS	4 WS	Difference(WS-DS)
Solar radiation	y = 0.0065x +21.795	0.0024	0.20	18.97±2.66	13.57±0.93	−5.40**
Sunshine	y = 0.0077x +5.8944	0.0254	0.23	8.67±2.25	5.47±0.88	−3.20**
Rainfall	y = 0.0047x +5.4759	0.0014	0.14	3.68±5.84	5.45±1.5	1.77
Evaporation	y = −0.0011x +4.576	0.0014	−0.03	6.13±1.13	3.66±0.26	−2.47**
Max. temp	y = 0.0132x +30.469	0.1456	0.40	32.7±1.06	30.85±0.28	−1.85**
Min. temp	y = 0.0404x +22.944	0.7231**	1.21	24.19±0.27	24.48±0.26	0.29
Mean temp	y = 0.0268x +26.7060	0.5811**	0.80	28.44±0.55	27.67±0.08	−0.77**
Max.-Min.temp	y = −0.0272x +7.5251	0.3793**	−0.82	8.51±1.09	6.37±0.52	−2.14**
VPD^a^	y = 0.0016x +0.1485	0.1570*	0.04	0.58±0.18	0.47±0.08	−0.11
RH^a^	y = −0.0725x +95.8310	0.1770*	−1.74	84.44±4.12	87.02±1.79	2.58

* ,** Significant at *P*<0.05 and *P*<0.01 probability level. VPD and RH represent vaporpressure deficit and relative humidity respectively.

a : data of VPD and RH for analysis was from 1980 to 2003 since new method applied after 2004.

### Difference in Weather Parameters between DS and WS During Grain Filling

Table 1 shows the average climate data for the complete grain-filling stage over the eight seasons. Over the four DS, the maximum temperature was higher than in the four WS, and the average minimum temperature during grain-filling in the four DS was slightly lower than in the four WS (Table 1), leading to a significantly larger diurnal variation in the DS compared to the WS. In the DS, radiation, sunshine and evaporation were significantly higher than in the WS (Table 1). Rainfall in the WS was also 1.48 times greater than in the DS (Table 1).

From flowering to harvest, the minimum temperature in the DS increased from an average of 23.82°C to 24.75°C, whereas in the WS, the minimum temperature ranged only from 24.45°C to 24.51°C. In most 5-day intervals after flowering, the difference in radiation, sunshine and evaporation between the DS and WS was significant (Table 2). No significant difference was observed in the 5 day-average for VPD or RH (Table 2).

**Table 2:** 5-day average of climatic data over 39 varieties in DS and WS during grain filling.

Climate	Season	0–5	6–10	11–15	16–20	21–25	26–30
Solar radiation	DS	19.30±1.71	18.87±3.12	19.03±3.50	18.60±3.33	18.95±2.25	19.09±2.19
	WS	14.21±0.50	13.46±1.04	13.38±1.71	13.73±1.16	13.53±0.82	13.10±1.14
	Difference	5.09**	5.41**	5.65**	4.87**	5.42**	5.99**
Sunshine	DS	8.73±1.56	8.57±2.54	8.61±2.92	8.47±2.75	8.75±2.14	8.89±1.64
	WS	5.61±0.90	5.18±1.16	5.23±1.19	5.61±1.11	5.69±0.93	5.50±0.84
	Difference	3.12**	3.39*	3.38*	2.86*	3.06**	3.39**
Rainfall	DS	3.33±4.99	4.84±8.25	5.17±9.50	3.33±5.65	2.48±3.42	2.96±3.49
	WS	5.99±2.48	5.32±1.19	4.87±2.11	5.48±1.53	5.07±1.84	5.97±1.32
	Difference	−2.66	−0.48	0.30	−2.15	−2.59	−3.01
Evaporation	DS	6.12±0.63	6.05±1.23	6.2±1.27	6.01±1.38	6.14±1.21	6.27±1.14
	WS	3.82±0.21	3.54±0.30	3.64±0.39	3.66±0.29	3.65±0.31	3.63±0.32
	Difference	2.30**	2.51**	2.56**	2.35**	2.49**	2.64**
Max. temp	DS	32.27±0.36	32.39±0.71	32.51±1.17	32.67±1.69	33.03±1.47	33.31±1.27
	WS	31.16±0.22	30.98±0.20	30.84±0.46	30.89±0.48	30.71±0.30	30.52±0.51
	Difference	1.11**	1.41**	1.67**	1.78*	2.32**	2.79**
Min. temp	DS	23.82±0.58	23.90±0.38	23.88±0.37	24.26±0.44	24.52±0.38	24.75±0.24
	WS	24.50±0.40	24.47±0.38	24.51±0.23	24.49±0.22	24.45±0.18	24.45±0.21
	Difference	−0.68*	−0.57*	−0.63*	−0.23	0.07	0.30*
Mean temp	DS	28.05±0.4	28.15±0.34	28.2±0.59	28.46±0.96	28.78±0.86	29.03±0.74
	WS	27.83±0.27	27.72±0.14	27.68±0.21	27.69±0.14	27.58±0.07	27.49±0.2
	Difference	0.22	0.43*	0.52	0.77	1.20**	1.54**
Max.- Min.temp	DS	8.45±0.53	8.49±0.92	8.63±1.27	8.41±1.57	8.52±1.28	8.56±1.07
	WS	6.67±0.35	6.51±0.53	6.34±0.59	6.40±0.7	6.26±0.48	6.07±0.68
	Difference	1.78**	1.98**	2.29**	2.01*	2.26**	2.49**
VPD	DS	0.56±0.13	0.57±0.13	0.56±0.16	0.58±0.21	0.60±0.22	0.60±0.25
	WS	0.46±0.07	0.45±0.07	0.48±0.08	0.46±0.10	0.46±0.09	0.49±0.07
	Difference	0.10	0.12	0.08	0.12	0.14	0.11
RH	DS	84.49±3.30	84.39±3.27	84.73±3.72	84.3±4.60	84.24±4.90	84.50±5.56
	WS	87.29±1.49	87.58±1.66	86.76±1.89	87.24±2.38	87.13±2.13	86.11±1.54
	Difference	−2.8	−3.19*	−2.03	−2.94	−2.89	−1.61

* , **represent significant at *P*<0.05 and *P*<0.01 probability level, respectively.

### Milling Quality

**Figure 1:**
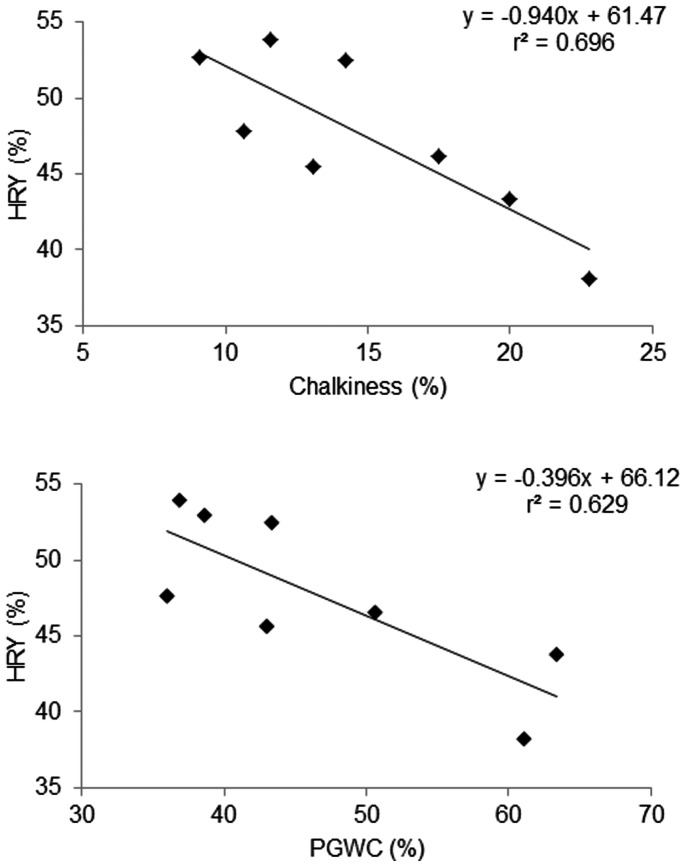
Association between averageof chalkiness, PGWC and HRY of 39 varieties across 8 seasons.

All varieties tested had long, slender grains in order to eliminate potential effects of grain shape on head rice yield and chalkiness. The average milling quality of each variety across the seasons is shown in Table S1. Across all seasons, chalkiness was lower in the DS compared to the WS, with averages of 13.10% and 16.63% respectively (Table 3).The difference was not significant, but the trend was observed every year. HRY in the WS was significantly lower than that in the DS (*P*<0.01). A strong negative relationship was found between average chalkiness and HRY over the seasons (*P*<0.01) and a negative relationship between PGWC and HRY (*P*<0.05; Fig. 1A and B). Across the set and seasons, HRY declined by ≈1% for each 1% increase in chalkiness. This negative trend was observed for 35 varieties. For the other four, the relationship between chalkiness and HRY was positive but not significant (Table S1).

**Table 3:** Averageof chalk and milling quality over 39 varieties in each season.

Season	Chalkiness (%)	PGWC (%)	HRY (%)
2005DS	9.09±5.40	38.62±21.19	52.96±8.86
2005WS	13.05±7.84	42.99±22.63	45.64±12.09
2006DS	18.00±13.77	50.60±27.06	46.56±13.28
2006WS	22.79±13.36	61.06±26.61	38.23±15.18
2007DS	11.45±7.59	36.86±21.90	53.95±7.76
2007WS	19.86±8.95	63.35±20.46	43.79±11.40
2008WS	10.88±7.34	35.99±21.81	47.64±6.12
2009DS	14.10±8.97	43.34±23.3	52.47±5.52
*LSD_0.05_*	8.90	13.46	7.92
*LSD_0.01_*	11.70	17.70	10.41
DS Average	13.10±3.60	42.38±5.72	51.31±3.46
WS Average	16.63±5.69	50.70±13.62	43.69±4.18
*t*-Value	−1.48	−1.59	3.97**

** represents significant at *P*<0.01.

### Effects of Climatic Parameters on Quality

Significant effects of several weather elements on grain quality were detected using the set of average values across the whole grain-filling stage (Table 4). Throughout grain-filling, the average VPD was negatively associated with chalkiness (*P*<0.05) and positively with HRY (*P*<0.05), while the average RH showed opposite associations with each trait. Radiation, evaporation and maximum temperature were positively associated with HRY (*P*<0.1). There was no significant relationship observed between other climate parameters and chalkiness in spite of several differences between the DS and the WS.

**Table 4:** Correlation coefficients between chalkiness, PGWC, HRY and climatic parameters during grain filling.

Trait	DAF	Solar Radiation	Sunshine	Rainfall	Evaporation	Max. temp	Min. temp	Mean temp	Max.-Min. temp	VPD	RH
Chalkiness	0–5	−0.444	−0.321	−0.066	−0.432	−0.377	0.329	−0.080	−0.422	−0.750*	0.709*
	6–10	−0.405	−0.299	0.034	−0.398	−0.451	0.161	−0.501	−0.378	−0.767*	0.727*
	11–15	−0.473	−0.390	0.023	−0.407	−0.539	0.192	−0.573	−0.482	−0.800**	0.782*
	16–20	−0.314	−0.188	0.030	−0.311	−0.365	−0.228	−0.412	−0.300	−0.724*	0.717*
	20–25	−0.307	−0.181	0.057	−0.301	−0.407	−0.643+	−0.482	−0.312	−0.664+	0.646+
	26–30	−0.381	−0.309	0.171	−0.313	−0.406	−0.778*	−0.471	−0.321	−0.612+	0.573
	0–30	−0.395	−0.289	0.035	−0.362	−0.437	−0.060	−0.498	−0.371	−0.725*	0.708*
PGWC	0–5	−0.438	−0.362	−0.122	−0.457	−0.300	0.377	0.037	−0.400	−0.556	0.538
	6–10	−0.445	−0.363	0.024	−0.444	−0.421	0.245	−0.400	−0.387	−0.616+	0.595+
	11–15	−0.512	−0.455	0.035	−0.456	−0.509	0.324	−0.478	−0.496	−0.622+	0.616+
	16–20	−0.342	−0.259	0.042	−0.340	−0.311	0.005	−0.306	−0.300	−0.549	0.554
	20–25	−0.325	−0.232	0.092	−0.314	−0.356	−0.430	−0.400	−0.296	−0.504	0.498
	26–30	−0.403	−0.364	0.161	−0.332	−0.384	−0.619+	−0.429	−0.323	−0.438	0.402
	0–30	−0.420	−0.349	0.032	−0.393	−0.394	0.114	−0.405	−0.370	−0.550	0.544
HRY	0–5	0.674*	0.485	−0.002	0.717*	0.682*	−0.474	0.249	0.695*	0.719*	−0.693*
	6–10	0.549	0.396	0.136	0.607+	0.602+	−0.419	0.524	0.623+	0.766*	−0.744*
	11–15	0.580	0.433	0.180	0.594+	0.601+	−0.543	0.494	0.632+	0.676*	−0.678*
	16–20	0.452	0.275	0.005	0.491	0.409	−0.098	0.382	0.415	0.705*	−0.710*
	20–25	0.562	0.365	−0.132	0.527	0.549	0.402	0.574	0.504	0.710*	−0.701*
	26–30	0.637+	0.540	−0.294	0.564	0.615+	0.751*	0.656+	0.557	0.636+	−0.579
	0–30	0.583+	0.420	0.027	0.586+	0.582+	−0.250	0.576	0.564	0.714*	−0.704*

+ , *and **represent significant at *P*<0.1, *P*<0.05 and *P*<0.01 probability level.

In order to understand the effects of weather elements on different stages of grain filling, the whole filling period was dissected into six stages of 5 days per stage (Table 4). High RH and low VPD during almost the whole grain-filling period were strongly associated with high chalkiness. Minimum temperature had a significant negative association with chalkiness during the late wax ripening and yellow ripening stage. RH and VPD, during most of grain-filling, were also the key factors that influenced HRY; lower RH and higher VPD led to higher HRY. Diurnal temperature had a significant positive association with HRY from flowering to 15 DAF. In addition, higher evaporation and maximum temperature during initial filling led to higher HRY. Minimum and mean temperature influenced HRY significantly at the yellow ripening stage with positive effects. High solar radiation at the initial filling and yellow ripening stage was also associated with increased HRY.

**Figure 2:**
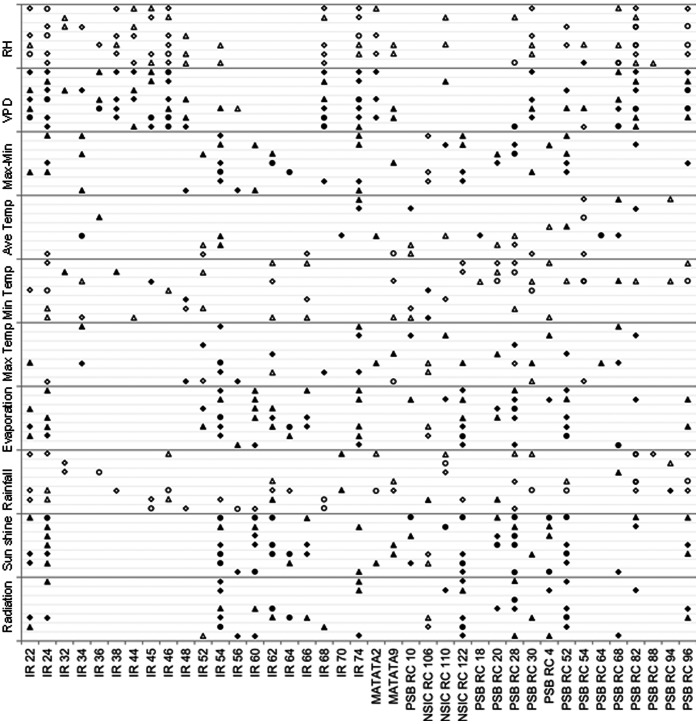
Association between HRY and weather parameters for each variety across 8 seasons. Triangles, diamonds and circles represent significance at *P*<0.10, *P*<0.05 and *P*<0.01 respectively. Blank and solid shapes represent negative and positive associations respectively. Gray lines from below to upper for each weather parameter represent 0–5, 6–10, 11–15, 16–20, 21–25, 26–30 and the whole grain filling stage.

**Figure 3:**
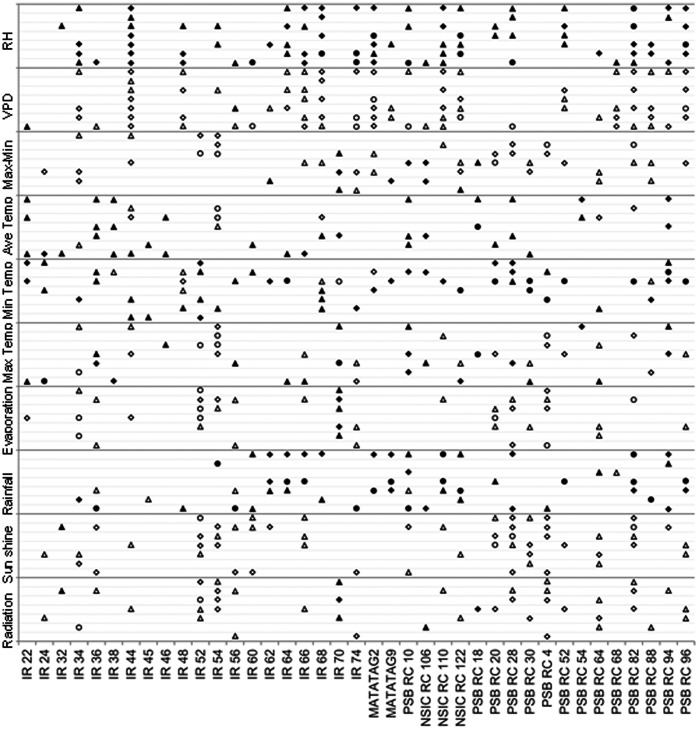
Association between chalkiness and weather parameters on single variety across 8 seasons. Triangles, diamonds and circles represent significance at *P*<0.10, *P*<0.05 and *P*<0.01 respectively. Blank and solid shapes represent negative and positive associations respectively. Gray lines from below to upper for each weather parameter represent 0–5, 6–10, 11–15, 16–20, 21–25, 26–30 and the whole grain filling stage.

**Figure 4:**
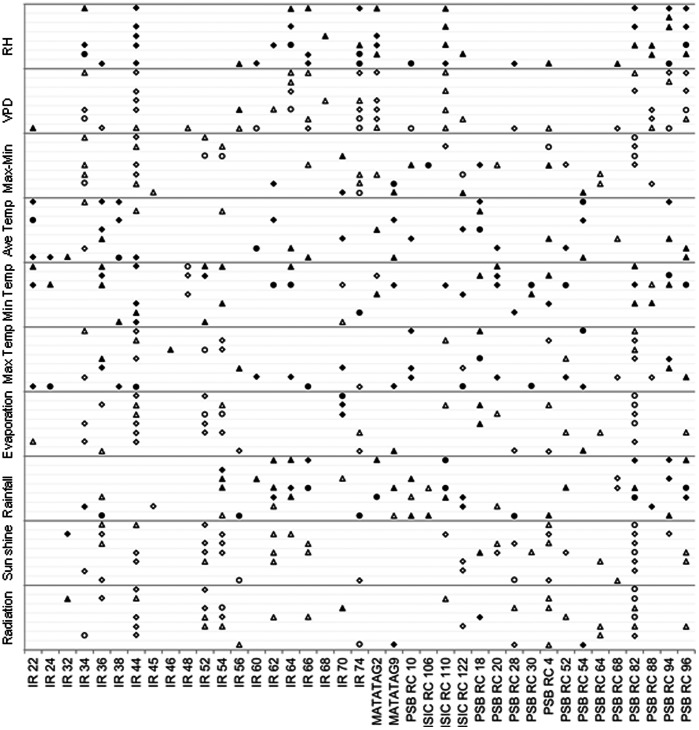
Association between PGWC and weather parameters for each variety across 8 seasons. Triangles, diamonds and circles represent significance at *P*<0.10, *P*<0.05 and *P*<0.01 respectively. Blank and solid shapes represent negative and positive associations respectively. Gray lines from below to upper for each weather parameter represent 0–5, 6–10, 11–15, 16–20, 21–25, 26–30 and the whole grain filling stage.

The same analysis as described above was done for weather parameters and HRY on each variety (Fig. 2).The trends detected for 23 of the 39 varieties matched the trends described above (Fig. 2). The relationship between maximum temperature and HRY seemed be specific to certain varieties. For example, the correlation at 6–15 DAF was positive for IR 54 but negative for NSIC RC 106 at the same stage (Fig. 2).The associations between chalkiness, PGWC and climate parameters were similar to those found across the set (Fig. 3, Fig. 4).RH and VPD were the main factors affecting rice appearance quality but with opposite direction. Higher radiation and, sunshine caused lower chalkiness and PGWC generally.

## Discussion

Recent research has shown that the mean global surface air temperature has increased over the past century, and other reports indicate that this will have its greatest impact in the rice-producing countries of Asia [4]. In the present paper, we show, as others have shown, that there has been a significant increase in the minimum and mean temperature, accompanied by a significant decrease in diurnal variation over the past 30 years at Los Baños in the Philippines (Table 1).

Higher temperature during grain-filling has been shown to affect rice quality in a number of studies [12], [15], [16], [17], [18], [19], [20]. High temperature during the early stages of grain-filling leads to an increase in the rate of endosperm cell division with the result that cell walls are thin, fewer granules are initiated, and the deposition of starch, on fewer building blocks within thin-walled cells, leads to higher PGWC and large chalky areas [21], [22]. High temperatures during the night have also been shown to lead to higher chalk values [19]. In some studies using controlled growth conditions, temperature was the only variable measured, leading to a general conclusion that temperature is the main factor affecting chalkiness and HRY. In the present study, using four years of field-grown rice, we show that maximum temperature increased over the whole grain-filling stage in the DS, and decreased over grain-filling in the WS (Table 2), but in contrast to other studies, the average chalkiness and the PGWC over the four years were both significantly and reproducibly higher in the cooler WS than in the hotter DS (Table 2). Moreover, we show that the association between chalkiness and temperature is not significant at most of the stages of grain-filling (Table 3).

HRY was lower in the WS than in the DS over the four years (Table 3). Furthermore, the difference in maximum temperature over grain-filling between the 2005DS, with the lowest chalkiness and 2^nd^ highest HRY, and 2006WS, which had the highest chalkiness and lowest HRY, was close to three degrees (Table S2), while minimum temperature was similar. Taken together, these data demonstrate (i) that the environmental conditions of the WS lead to fewer and less valuable whole grains, than in the DS, (ii) that temperature alone is unlikely to be the major factor that leads to higher chalk and lower HRY, and (iii) other climate elements, or interactions between them, play a more significant role than temperature on the postharvest yield and quality of rice.

Solar radiation and sunshine were significantly different between the wet and dry seasons over four years (Table 1, Table 2), and the main climate parameters that associated with chalkiness in the field conditions over the four years were VPD, which associated negatively, and RH which associated positively (Table 4). Both of these factors influence transpirational capacity and therefore [23]. Both of these parameters correlated with chalkiness at every stage of grain-filling, and they also correlated throughout grain-filling, and in the opposite directions, with HRY (Table 4). A further demonstration of this is shown in Table S2, which compares climate factors during grain-filling in the 2005DS and 2006WS. Average chalk in the 2006WS was the highest of the dataset, at 22.79%, and in the 2005DS it was the lowest at 9.09% (Table 3). HRY was also about 15 percentage points higher in the 2005DS compared with the 2006WS. In these two seasons, the minimum and mean temperatures were similar throughout grain-filling (Table S2). However solar radiation, sunshine, evaporation and VPD were all substantially lower in the 2006WS, and rainfall and RH were higher, perhaps leading to less capacity for transpirational cooling to take place.

Transpirational cooling has a significant effect on the temperature of the canopy, and it is also linked with the dynamics of sucrose transport from sources in leave and stem to sinks, such as the developing endosperm, with significant implications for yield [24]. Within the grain, the phenotype of chalk appears as incompletely filled starch granules [8]. If capacity for transpirational cooling affects yield, then it affects the allocation of substrate to the developing panicle, and therefore, most likely affects chalkiness as well. Atmospheric VPD follows a diurnal pattern, being lowest at sunrise and increasing to a maximum at around 15:00 [25], where it would create the greatest moisture potential, leading to stomatal opening and transpiration. Dry matter accumulation follows a linear relationship withincreasing VPD under both controlled and uncontrolled climatic conditions at IRRI [26], further supporting the importance of transpirational cooling for the development of grains that are densely packed with well-developed and mature starch granules.

Chalkiness is the not the only parameter affected by climate in the WS; HRY is also affected. Figure 1 shows clearly that chalkiness and HRY are closely related, suggesting that the capacity for transpirational cooling is necessary also to minimize post-harvest losses of grain. Since chalkiness and HRY are the two factors that are used to determine the price of rice on both domestic and international markets, transpirational cooling appears to be an ecessary trait for maximal value of the rice market chain. The data suggest that global warming *per se* might not be the major factor that leads to losses in yield and quality of rice, but the consequences of global warming, together with the climate of the WS, lead to higher RH and lower VPD, which lead to decreased evaporation and therefore capacity for transpirational cooling.

Table 2 shows that over four years, radiation, sunshine, VPD and evaporation are higher, and rainfall and humidity are lower in the dry seasons compared with the wet seasons, further indicating the antagonistic effect of global warming in a WS climate, whereas global warming could have a synergistic effect in a DS environment, by increasing VPD and consequently transpirational cooling, enabling the canopy to remain cool despite the higher air temperature.

The data in the present paper suggest that new, climate-ready varieties of rice should carry allelic combinations that lead to increased capacity for transpirational cooling, especially those that are grown in the wet season, in order to obtain the maximum number of whole and high quality grains. Variability for this trait has already been demonstrated in rice [24] providing the phenotypes that can be used to discover the best alleles for this trait.

All varieties used in the present study were developed by IRRI and have been widely grown in South and South East Asia over the past 30 years. The relationships between quality traits and weather parameters analyzed based on average data reflect the overall trend about how climate change affects rice grain quality. Results of single variety analysis could guide breeders on parental selection for progeny resistant to climate change. It is very interesting that very few significant associations were detected between HRY and weather parameters in some varieties (Fig. 2). Among these, the low chalk varieties such as IR 60, IR 64, PSB RC 88 and PSB RC 94 gave high HRY in both DS and WS, provided a set of potential parent lines in breeding programs targeting grain quality.

### Conclusion

The single parameter of high temperature does not lead to chalk under natural field conditions. The present study shows clearly, that in field conditions, the primary factors leading to chalk are a combination of climatic parameters that lead to conditions unfavorable for evaporation and transpirational cooling, and these differ for the wet and dry season. In the DS, global warming is likely to have synergistic effects for the major physical parameters of rice – chalk and HRY, but combined with the climate of the WS, the effects will be antagonistic, lowering HRY and increasing chalk.

## Supporting Information

Table S1 Average of flowering day and milling quality across seasons.
(DOCX)

Table S2 Weather data of grain filling stage on 2005DS and 2006WS.
(DOCX)
