# Supplementary material for: Climate Change: Implications for the Yield of Edible Rice
Source: PLoS One. 2013 Jun 12;8(6):e66218. doi: 10.1371/journal.pone.0066218 (PMC3680399; doi:10.1371/journal.pone.0066218)
Supplement: Table S1 — Average of flowering day and milling quality across seasons. (DOCX) [file pone.0066218.s001.docx]

Supporting Table 1 Average of flowering day and milling quality across seasons

| Varieties | Flowering day | | GL  (mm) | GW  (mm) | GL/  GW | Chalkiness (%) | | PGWC (%) | | HRY (%) | | Correlation coefficients^b^ | |
| --- | --- | --- | --- | --- | --- | --- | --- | --- | --- | --- | --- | --- | --- |
|  | DS | WS |  |  |  | DS | WS | DS | WS | DS | WS | Chalkiness | PGWC |
| IR 22 | 89±2^a^ | 93±1 | 6.44 ± 0.09 | 1.99 ± 0.09 | 3.24 ± 0.09 | 3.2±3.4 | 1.5±0.7 | 14.0±15.4 | 4.7±3.7 | 57.5±3.4 | 46.8±14.9 | -0.152 | -0.157 |
| IR 24 | 96±2 | 85±1 | 6.50 ± 0.15 | 2.11 ± 0.05 | 3.08 ± 0.10 | 5.0±0.9 | 7.8±4.5 | 19.8±3.9 | 27.9±20.0 | 52.4±8.6 | 42.4±7.5 | -0.548 | -0.422 |
| IR 32 | 105±2 | 105±2 | 6.44 ± 0.06 | 2.06 ± 0.02 | 3.13 ± 0.04 | 25.7±15.7 | 14.3±11.8 | 73.4±30 | 48.3±29.5 | 40.1±8.2 | 42.2±2.9 | -0.195 | -0.075 |
| IR 34 | 96±2 | 92±4 | 6.60 ± 0.10 | 2.12 ± 0.06 | 3.11 ± 0.08 | 4.3±1.5 | 10.8±3.3 | 14.4±6.9 | 41.9±17.5 | 53.6±9.5 | 42.8±9.2 | -0.315 | -0.394 |
| IR 36 | 83±2 | 80±1 | 6.43 ± 0.07 | 2.02 ± 0.07 | 3.18 ± 0.07 | 10.8±10.8 | 17.0±13.6 | 30.6±28.1 | 48.0±28.9 | 46.6±4.9 | 44.2±11.9 | 0.413 | 0.383 |
| IR 38 | 93±4 | 92±1 | 6.35 ± 0.03 | 2.07 ± 0.03 | 3.07 ± 0.03 | 32.7±11.4 | 31.0±3.7 | 79.2±9.1 | 78.9±3.0 | 48.2±17.9 | 41.1±18.5 | -0.626^+^ | -0.478 |
| IR 44 | 99±3 | 95±0 | 6.57 ± 0.04 | 2.10 ± 0.03 | 3.13 ± 0.04 | 10.0±3.7 | 14.5±5.0 | 33.5±7.0 | 49.7±11.5 | 45.7±17.7 | 34.3±11.1 | -0.819^**^ | -0.775^*^ |
| IR 45 | 95±3 | 90±2 | 6.48 ± 0.04 | 2.18 ± 0.04 | 2.97 ± 0.04 | 29.7±6.8 | 28.5±5.9 | 80.2±6.7 | 79.9±12.8 | 37.9±17.8 | 34.5±19.1 | 0.128 | 0.354 |
| IR 46 | 94±3 | 92±1 | 6.31 ± 0.04 | 2.03 ± 0.04 | 3.11 ± 0.04 | 11.7±9.8 | 13.0±2.8 | 34.9±28.1 | 30.8±7.3 | 46.9±7.2 | 40.3±15.3 | -0.268 | -0.213 |
| IR 48 | 99±5 | 97±2 | 6.30 ± 0.03 | 2.05 ± 0.04 | 3.07 ± 0.04 | 8.1±1.9 | 8.7±4.0 | 24.7±4.1 | 23.5±9.6 | 46.8±11.3 | 35.1±14.2 | -0.630^+^ | -0.332 |
| IR 52 | 86±1 | 76±0 | 6.65 ± 0.09 | 2.00 ± 0.03 | 3.33 ± 0.06 | 6.2±3.0 | 10.3±6.7 | 19.3±8.7 | 37.6±25.0 | 43.6±12.1 | 28.7±12.2 | -0.661^+^ | -0.810^**^ |
| IR 54 | 91±2 | 93±1 | 6.41 ± 0.14 | 2.00 ± 0.05 | 3.21 ± 0.10 | 12.3±8.1 | 14.7±4.7 | 41.9±16.3 | 44.6±8.3 | 56.2±1.6 | 38.3±6.0 | -0.233 | -0.187 |
| IR 56 | 82±2 | 74±1 | 6.56 ± 0.05 | 2.02 ± 0.04 | 3.25 ± 0.05 | 7.0±2.7 | 8.5±3.9 | 25.7±10.2 | 31.2±20.0 | 49.7±4.4 | 43.8±13.3 | -0.691^*^ | -0.775^*^ |
| IR 60 | 82±1 | 78±2 | 6.34 ± 0.03 | 1.91 ± 0.02 | 3.32 ± 0.03 | 3.6±1.3 | 4.5±1.7 | 12.5±3.7 | 14.3±4.9 | 61.5±1.9 | 52.2±8.5 | -0.587^+^ | -0.430 |
| IR 62 | 84±2 | 88±2 | 6.24 ± 0.04 | 2.11 ± 0.04 | 2.96 ± 0.04 | 9.8±4.5 | 8.3±4.0 | 31.7±13.4 | 32.1±16.4 | 54.5±4.0 | 40.4±13.9 | -0.688^*^ | -0.747^*^ |
| IR 64 | 84±0 | 82±2 | 6.66 ± 0.08 | 2.01 ± 0.04 | 3.31 ± 0.06 | 12.4±2.4 | 19±14.7 | 51.1±14.7 | 65.6±31.3 | 61.0±3.9 | 53.8±3.5 | -0.264 | -0.407 |
| IR 66 | 82±1 | 78±0 | 6.41 ± 0.03 | 1.93 ± 0.03 | 3.32 ± 0.03 | 3.8±1.1 | 9.5±8.2 | 17.1±4.0 | 31.0±24.5 | 60.7±2.2 | 52.9±6.8 | -0.059 | 0.010 |
| IR 68 | 95±2 | 91±1 | 7.01 ± 0.06 | 2.08 ± 0.03 | 3.37 ± 0.05 | 16.8±6.3 | 16.5±7.9 | 51.1±13.8 | 46.9±10.9 | 46.3±6.4 | 39.5±14.5 | -0.521 | -0.312 |
| IR 70 | 93±5 | 89±5 | 6.49 ± 0.11 | 2.04 ± 0.03 | 3.18 ± 0.07 | 16.8±6.5 | 17.3±7.1 | 57.9±9.8 | 57.8±22.0 | 42.4±7.4 | 49.2±11.7 | 0.414 | 0.529 |
| IR 74 | 102±2 | 97±0 | 6.66 ± 0.10 | 2.03 ± 0.03 | 3.28 ± 0.07 | 14.6±4.5 | 22±8.2 | 48.9±9.1 | 66.7±14.3 | 48.8±6.7 | 35.1±4.8 | -0.556 | -0.701^*^ |
| MATATAG2 | 91±1 | 87±1 | 6.69 ± 0.07 | 1.82 ± 0.07 | 3.68 ± 0.07 | 8.3±4.5 | 13.5±13.8 | 31.2±17.1 | 42.7±32.9 | 52.4±6.5 | 41.7±10.4 | -0.732^*^ | -0.721^*^ |
| MATATAG9 | 84±1 | 87±1 | 6.54 ± 0.08 | 1.97 ± 0.03 | 3.32 ± 0.06 | 15.6±2.9 | 19.0±20.0 | 55.4±7.7 | 49.8±38.9 | 43.4±4.6 | 36.7±10.0 | -0.815^**^ | -0.792^*^ |
| NSIC RC 106 | 82±1 | 79±1 | 6.36 ± 0.05 | 2.12 ± 0.05 | 3.00 ± 0.05 | 20.0±4.3 | 25±12.1 | 65.1±6.0 | 67.9±25.2 | 56.7±5.8 | 49.4±6.9 | -0.698^*^ | -0.490 |
| NSIC RC 110 | 86±2 | 87±1 | 6.49 ± 0.07 | 2.02 ± 0.03 | 3.21 ± 0.05 | 2.1±0.1 | 8.0±2.7 | 6.8±3.2 | 28.3±9.0 | 49.4±6.4 | 38.1±16.2 | -0.356 | -0.427 |
| NSIC RC 122 | 94±2 | 89±4 | 6.83 ± 0.07 | 2.05 ± 0.03 | 3.33 ± 0.05 | 12.9±0.1 | 13.8±4.9 | 46.6±0.7 | 43.0±17.1 | 35.8±8.7 | 43.7±13.9 | -0.289 | -0.530 |
| PSB RC 10 | 79±3 | 73±0 | 6.33 ± 0.05 | 2.06 ± 0.04 | 3.07 ± 0.05 | 8.3±3 | 15.3±14.7 | 32.2±10.8 | 43.5±30.5 | 59.1±7.4 | 57.0±5.3 | -0.308 | -0.266 |
| PSB RC 18 | 90±2 | 102±2 | 6.52 ± 0.05 | 2.15 ± 0.03 | 3.03 ± 0.04 | 23.0±9.6 | 19.5±14.5 | 69.7±21.3 | 60.7±24.1 | 53.3±10.9 | 46.9±16.1 | 0.087 | 0.139 |
| PSB RC 20 | 83±3 | 79±0 | 6.55 ± 0.06 | 1.92 ± 0.03 | 3.41 ± 0.05 | 6.6±1.6 | 13.8±5.6 | 28.1±5.8 | 42.1±20.3 | 56.7±5.6 | 42.3±13.3 | -0.974^**^ | -0.948^**^ |
| PSB RC 28 | 83±2 | 77±0 | 6.43 ± 0.05 | 2.00 ± 0.03 | 3.22 ± 0.04 | 14±6.1 | 20.0±7.4 | 56.5±13.5 | 65.7±12.7 | 60.9±2.6 | 46.8±10.5 | -0.859^**^ | -0.767^*^ |
| PSB RC 30 | 89±2 | 84±0 | 6.57 ± 0.05 | 2.09 ± 0.02 | 3.14 ± 0.04 | 13.1±0.1 | 18.8±5.7 | 51.6±8.6 | 63.3±20.1 | 49.8±6.0 | 38.5±14.3 | -0.786^*^ | -0.695^*^ |
| PSB RC 4 | 81±2 | 72±0 | 6.45 ± 0.06 | 1.98 ± 0.05 | 3.26 ± 0.06 | 4.4±1.3 | 10.5±3 | 19.4±5.6 | 40.0±18.4 | 60.2±3.8 | 54.1±7.4 | -0.571 | -0.523 |
| PSB RC 52 | 85±1 | 86±0 | 6.32 ± 0.03 | 1.94 ± 0.02 | 3.26 ± 0.03 | 8.0±4.0 | 20±12.5 | 32.0±11.9 | 58.5±30.7 | 58.1±1.6 | 46.5±4.9 | -0.653^+^ | -0.566 |
| PSB RC 54 | 85±4 | 79±5 | 6.53 ± 0.19 | 2.04 ± 0.04 | 3.20 ± 0.12 | 26.1±19 | 15.0±8.0 | 67.8±23.3 | 50.3±23.4 | 49.5±9.3 | 48.1±15.2 | -0.555 | -0.525 |
| PSB RC 64 | 96±2 | 95±0 | 6.83 ± 0.07 | 2.07 ± 0.04 | 3.30 ± 0.06 | 14.0±2.9 | 19.3±8.2 | 45.2±8.3 | 61.0±21.7 | 49.2±7.1 | 46.9±10.6 | -0.237 | -0.315 |
| PSB RC 68 | 89±2 | 99±1 | 6.90 ± 0.06 | 2.19 ± 0.04 | 3.15 ± 0.05 | 24.3±14.1 | 28.3±10.2 | 68.2±24.8 | 78.4±17.4 | 46.5±9.5 | 33.7±14.8 | -0.767^*^ | -0.765^*^ |
| PSB RC 82 | 84±1 | 89±1 | 6.65 ± 0.06 | 2.04 ± 0.03 | 3.26 ± 0.05 | 21.1±5.8 | 30.8±11.2 | 67.9±8.9 | 89.0±6.8 | 52.5±8.1 | 45.8±14.7 | -0.929^**^ | -0.660^+^ |
| PSB RC 88 | 93±1 | 88±2 | 6.32 ± 0.03 | 2.06 ± 0.03 | 3.07 ± 0.03 | 9.6±6.5 | 21.5±20.5 | 42.1±22.0 | 60.2±27.5 | 59.2±7.2 | 56.6±8.0 | -0.420 | -0.188 |
| PSB RC 94 | 84±3 | 76±0 | 6.42 ± 0.10 | 2.01 ± 0.05 | 3.19 ± 0.08 | 12.8±8.7 | 15.8±7.5 | 41±19.8 | 52.3±27.9 | 55.2±5.4 | 54.3±4.5 | -0.565 | -0.380 |
| PSB RC 96 | 82±1 | 75±0 | 6.61 ± 0.11 | 1.96 ± 0.03 | 3.37 ± 0.07 | 14.5±5.8 | 26.3±15.6 | 49.2±10.3 | 69±27.3 | 51.7±4.1 | 43±10.0 | -0.967^**^ | -0.979^**^ |

^a^Mean ± standard error of the mean; ^b^ represents correlation coefficients between chalkiness, PGWC and HRY.

^+^, ^*^and ^**^represent significant at *P*<0.1, *P*<0.05 and *P*<0.01 probability level.
