# Supplementary material for: Climate Change: Implications for the Yield of Edible Rice
Source: PLoS One. 2013 Jun 12;8(6):e66218. doi: 10.1371/journal.pone.0066218 (PMC3680399; doi:10.1371/journal.pone.0066218)
Supplement: Table S2 — Weather data of grain filling stage on 2005DS and 2006WS. (DOCX) [file pone.0066218.s002.docx]

Supporting Table 2 Weather data of grain filling stage on 2005DS and 2006WS

| DAF | Season | Solar Radiation | Sun shine | Rainfall | Evaporation | Max. Temp | Min. Temp | Mean Temp | Max.-Min. | VPD | RH |
| --- | --- | --- | --- | --- | --- | --- | --- | --- | --- | --- | --- |
| 0-5 | 2005DS | 20.88 | 9.70 | 0.69 | 6.47 | 32.62 | 23.52 | 28.07 | 9.10 | 0.72 | 80.25 |
|  | 2006WS | 14.34 | 6.61 | 3.97 | 4.07 | 30.93 | 24.05 | 27.49 | 6.88 | 0.37 | 89.07 |
| 6-10 | 2005DS | 20.25 | 9.51 | 1.04 | 6.33 | 33.16 | 23.88 | 28.52 | 9.28 | 0.72 | 80.79 |
|  | 2006WS | 14.06 | 6.43 | 3.57 | 3.81 | 31.02 | 24.04 | 27.53 | 6.99 | 0.36 | 89.33 |
| 11-15 | 2005DS | 20.48 | 9.68 | 1.03 | 6.55 | 33.57 | 24.18 | 28.87 | 9.39 | 0.76 | 80.41 |
|  | 2006WS | 13.13 | 5.88 | 2.17 | 3.87 | 30.64 | 24.27 | 27.45 | 6.37 | 0.37 | 89.17 |
| 16-20 | 2005DS | 20.22 | 9.25 | 1.24 | 6.56 | 34.43 | 24.87 | 29.65 | 9.56 | 0.80 | 79.96 |
|  | 2006WS | 14.65 | 7.09 | 3.33 | 4.05 | 31.28 | 24.28 | 27.78 | 7.00 | 0.33 | 90.38 |
| 20-25 | 2005DS | 20.12 | 9.38 | 0.88 | 6.73 | 34.64 | 25.07 | 29.85 | 9.57 | 0.80 | 80.43 |
|  | 2006WS | 14.63 | 7.09 | 2.36 | 4.09 | 31.04 | 24.26 | 27.65 | 6.78 | 0.36 | 89.39 |
| 26-30 | 2005DS | 20.29 | 9.31 | 1.08 | 6.78 | 34.55 | 25.08 | 29.82 | 9.47 | 0.82 | 79.80 |
|  | 2006WS | 13.87 | 6.37 | 4.15 | 4.10 | 30.86 | 24.16 | 27.51 | 6.69 | 0.41 | 87.80 |
| 0-30 | 2005DS | 20.37 | 9.47 | 0.99 | 6.57 | 33.83 | 24.43 | 29.13 | 9.40 | 0.77 | 80.27 |
|  | 2006WS | 14.11 | 6.58 | 3.26 | 4.00 | 30.96 | 24.18 | 27.57 | 6.78 | 0.37 | 89.19 |
